# Fetal and Maternal Diseases in Pregnancy: From Morphology to Function

**DOI:** 10.3390/diagnostics12051117

**Published:** 2022-04-29

**Authors:** Gabriele Masselli

**Affiliations:** Radiology Department, Umberto I Hospital Sapienza University, 00161 Rome, Italy; gabriele.masselli@uniroma1.it

Ultrasound (US) is currently the standard approach for the initial evaluation of fetal anatomy and maternal conditions during pregnancy since it facilitates a real-time examination and is widely available and cost-effective.

Magnetic resonance imaging (MRI), due to its capabilities, can greatly improve the diagnostic performance of US during pregnancy [1,2]. 

The advances made in MRI techniques have overcome the technical difficulties of imaging a structure that is affected by both fetal and maternal motions.

In recent years, MRI has become a useful element in the decision-making process for fetal abnormalities and maternal diseases in pregnancy, proving to offer unequivocal advantages over ultrasound [1,2,3].

The added value of MRI-based imaging is three-fold, and leads to the following: (a) an improvement in diagnostic accuracy by adequate morphological examination, (b) the detection of additional anomalies, and (c) in addition, MRI has the potential to provide information regarding renal and pulmonary functions.

Fetal MR imaging using fast and ultrafast pulse sequences assists in acquisition of high-quality images regardless of maternal body habitus, fetal position, or amniotic fluid index. Fetal MRI is also not hampered by the acoustic shadowing from fetal pelvic bones, thereby facilitating multiplanar imaging and a better differentiation of kidneys and pelvic organs compared to US, especially late in pregnancy.

MRI can improve the management of many high-risk pregnancies such as those complicated by PAD, placental abruption, or acute abdominal pain; upgrades the counseling in different clinical conditions including a trial of vaginal birth after cesarean section or CMV infection; is useful in the recognition of the developing fetal anatomy and in the detection of subtle fetal abnormalities or complex lesions; and may guide therapy, particularly when perinatal intensive care and surgery are considered or when delivery is expected to present unique challenges [2,3,4].

Moreover, MRI offers a promising noninvasive method by which to interrogate multiple facets of placental development and function and to investigate the pathophysiology of severe disorders such as intrauterine growth restriction (IUGR) and preeclampsia (PIH) [3,4].

MRI provides excellent resolution, anatomical detail, multiplanar acquisition, and permits 3D reconstruction in normal and pathological settings [5].

With the advent of rapid sequences and short acquisition times, the use of fetal MRI has evolved exponentially. It has become key in the optimization of prenatal counseling, as it often refines or modifies the US based prenatal diagnosis, thereby altering the management of the pregnancy. MRI is particularly helpful in evaluating complex fetal abnormalities [2,3].

The role of fetal MRI is to add metabolic and functional studies, address the challenge of objectively detecting diffuse white matter lesions and early damage with the help of DWI and ADC mapping, and to visualize abnormal white matter tracts with prenatal tractography. Functional MRI can be a useful tool for evaluating blood oxygenation in human fetuses to monitor high risk fetuses and sensorial fetal activity [4,5].

Arterial spin labeling (ASL) and/or intravoxel incoherent motion (IVIM) have also been used to measure placental perfusion in pregnant women. ASL or IVIM could overcome the limitation of contrast agents as these imaging techniques do not require the use of an exogenous contrast agent [4].

Although the use of Gd-based contrast agents in pregnant women is currently very limited, DCE-based techniques remain a robust method to measure the in vivo perfusion of organs and our study paves the way and provides a benchmark for future developments in placental perfusion studies [4].

Functional placental MRI reveals a range of placental changes, associated with inflammatory processes that have been confirmed on subsequent histology. 

It shows promise as a tool to noninvasively identify inflammation in vivo, and could therefore assist in determining the optimal timing for interventions designed to prevent fetal injury, such as antenatal corticosteroids and magnesium sulfate and the need for delivery and/or in utero transfers where indicated [4,5].

It is hoped that MRI will permit a noninvasive, longitudinal estimation of the renal function. However, there are still many challenges to overcome, such as its validation and determining its technical limitations, as well as the need for compensation for both maternal and fetal motion and models that account for rapid changes with gestational age. This will require new tools for advanced image registration, segmentation, and model-fitting, which can only be developed through close cross-disciplinary collaboration and the sharing of data and software (5).

In conclusion, MRI provides a morphological and functional evaluation of fetal and placental diseases and these joined data can provide an early diagnosis and better management in many conditions.
